# Reviewed article: Azevedo SPF, Damião JJ, Castro IRR. Brazilian dietary guidelines: interfaces with the mother and child hospital context. Epidemiol Serv Saude. 2025:34;e20240391

**DOI:** 10.1590/S2237-96222024v34e20240391.c

**Published:** 2025-05-02

**Authors:** Bruna Teles Soares Beserra

**Affiliations:** UFMT

**Table:** 

Rating	Excellent	Good	Average	Below average	Poor
Interest	✓				
Quality		✓			
Originality	✓				
Overall		✓			

**Table:** 

Questionnaire	Yes	No	Not applicable
Does the manuscript contain new and significant information to justify publication?	✓		
Does the Abstract (Summary) clearly and accurately describe the content of the article?	✓		
Is the problem significant and concisely stated?	✓		
Are the methods described comprehensively?	✓		
Are the interpretations and conclusions justified by the results?	✓		
Is adequate reference made to other work in the field?	✓		

## Manuscript Structure:

Length of article is: Adequate

Number of tables is: Adequate

Number of figures is: Adequate

Please state any conflict(s) of interest that you have in relation to the review of this paper (state “none” if this is not applicable): None

## Comments

Prezados Autores!

O artigo “Guias alimentares brasileiros: interfaces com o contexto hospitalar materno-infantil”, tem qualidade cientifica e metodológica. No entanto, algumas pequenas alterações no manuscrito são sugeridas.

1. O manuscrito precisa de revisão cuidadosa, com atenção ao idioma português.

2. Na introdução, os autores comentaram sobre ausência de guias alimentares no contexto hospitalar. Já que é uma ausência de guias no contexto hospitalar de maneira geral, os autores poderiam justificar por meio da literatura porque restringiu a criação da matriz apenas para o público hospitalar materno-infantil.

3. Nos métodos, na secção etapas do estudo faltou colocar os locais que foram incluídos no estudo, os autores mencionaram apenas os locais que foram excluídos. Além disso, os autores poderiam descrever mais detalhadamente os especialistas que participaram do painel dos especialistas, eles apresentavam domínio teórico e/ou experiência prática em que assunto? Por que no primeiro painel teve a presença de 13 especialistas e no segundo apenas três especialistas?

4. Nos Resultados retirar a descrição dos espaços do ambiente hospitalar selecionado, isso faz parte dos métodos.

5. Na Discussão seria interessante também discutir a relevância da construção da matriz para o contexto materno-infantil de uma maneira mais específica, uma vez que o título do trabalho aborda esse ambiente hospitalar.

